# On the relationship between inhibition and receptor occupancy by nondepolarizing neuromuscular blocking drugs

**DOI:** 10.1186/s12976-021-00147-w

**Published:** 2021-08-21

**Authors:** Hikaru Hoshino, Eiko Furutani

**Affiliations:** 1 Department of Electrical Materials and Engineering, University of Hyogo, Hyogo, Japan; 2 Department of Anesthesiology, Kagawa University, Kagawa, Japan

**Keywords:** Anesthesia, Neuromuscular blockade, Receptor binding models, Nonlinear relationship

## Abstract

**Background:**

Nondepolarizing neuromuscular blocking drugs (NDNBs) are clinically used to produce muscle relaxation during general anesthesia. To better understand clinical properties of NDNBs, comparative in vitro pharmacologic studies have been performed. In these studies, a receptor binding model, which relies on the assumption that the inhibition, i.e., the effect of an NDNB, is proportional to the receptor occupancy by the drug, has been effectively used to describe obtained experimental data. However, it has not been studied in literature under which conditions the above assumption can be justified nor the assumption still holds in vivo. The purpose of this study is to explore the in vivo relationship between the inhibition and the receptor occupancy by an NDNB and to draw implications on how in vitro experimental results can be used to discuss the in vivo properties of NDNBs.

**Methods:**

An ordinary differential equation model is employed to simulate physiologic processes of the activation of receptors by acetylcholine (ACh) as well as inhibition by an NDNB. With this model, the degree of inhibition is quantified by the fractional amount of receptors that are not activated by ACh due to the presence of an NDNB. The results are visualized by plotting the fractional amounts of the activated receptors as a function of the receptor occupancy.

**Results:**

Numerical investigations reflecting in vivo conditions show that the degree of inhibition is not proportional to the receptor occupancy, i.e., there is a nonlinear relationship between the inhibition and the receptor occupancy. However, under a setting of high concentration of ACh reflecting a typical situation of in vitro experiments, the relationship between the inhibition and the receptor occupancy becomes linear, suggesting the validity of the receptor binding model. Also, it is found that the extent of nonlinearity depends on the selectivity of NDNBs for the two binding sites of the receptors.

**Conclusions:**

While the receptor binding model may be effective for estimating affinity of an NDNB through in vitro experiments, these models do not directly describe in vivo properties of NDNBs, because the nonlinearity between the inhibition and the receptor occupancy causes the modulation of the resultant concentration-effect relationships of NDNBs.

## Background

Nondepolarizing neuromuscular blocking drugs (NDNBs) inhibit neuromuscular transmission by competing with acetylcholine (ACh) for binding sites at the post-junctional nicotinic acetylcholine receptors (AChRs). They are widely used during general anesthesia to produce muscle relaxation for facilitating tracheal intubation and for providing optimal surgical working conditions [1]. To describe in vivo properties of NDNBs and to understand the molecular mechanisms behind clinical observations, many in vitro studies have been conducted (see e.g., [2–10]). In particular, in [7, 10], in vitro experiments have been conducted through macroscopic current recordings from outside-out patches of BOSC23 cells or Xenopus oocytes where human adult (rather than mouse adult or embryonic) muscle-type AChRs were expressed. In [7], synergistic effects of pairs of NDNBs have been studied. Although it successfully identified evidence for synergy between many pairs of NDNBs at the receptor level, not all the results correlated with synergism observed in vivo. In [9, 10], it was found that the I*C*_50_, the drug concentration needed to produce a 50*%* inhibition of the ACh-induced current, decreases with the increase in the concentration of ACh. Although it was concluded in [9, 10] that this demonstrated the existence of a noncompetitive action of NDNBs, some researchers raised concerns [11] over the insufficiency in quantitative analysis to draw the conclusion. Thus, more investigations and considerations are needed to describe the clinical observations and to clarify underlying mechanisms of inhibition based on in vitro experimental results.

While there are several measures identifying in vitro properties of NDNBs, potency [12] is one of the most widely used measures for studying NDNBs. It is usually quantified by I*C*_50_ estimated by regression analysis using the Hill equation for the relative current *I*_antag_/*I*_0_ given by

1 $$\begin{array}{*{20}l} \frac{I_{\text{antag}}}{I_{0}} = \frac{{\text{IC}_{50}}^{n_{\mathrm{H}}}}{{\text{IC}_{50}}^{n_{\mathrm{H}}} + {[\!\mathrm{D}]}^{n_{\mathrm{H}}}}, \end{array} $$

where *I*_0_ and *I*_antag_ stand for the currents measured in the absence and presence of the drugs, respectively. The parameter *n*_H_ stands for the Hill coefficient, and [ D] for the drug concentration. Also, affinity [12], which is defined as the extent or fraction of drug binding to receptors at any given drug concentration, is used for characterizing the strength of the binding of a ligand to its receptors. In [4–8], the relative current versus concentration curves were analyzed based on the two-site receptor binding model given by the following equation:

2 $$\begin{array}{*{20}l} \frac{I_{\text{antag}}}{ I_{0}} = \frac{K_{\mathrm{D1}} K_{\mathrm{D2}}}{ K_{\mathrm{D1}} K_{\mathrm{D2}} + K_{\mathrm{D1}} {[\!\mathrm{D}]} + K_{\mathrm{D2}} {[\!\mathrm{D}]} + {[\!\mathrm{D}]}^{2} },  \end{array} $$

where *K*_D1_ and *K*_D2_ stand for the dissociation equilibrium constants for NDNBs binding to the first and second sites of an AChR, respectively. Since the right-hand side of Eq. (2) represents the fraction of free receptors, i.e., the fraction of receptors that are not occupied by the drugs, the model (2) implies that the potency of an NDNB can be completely characterized by the affinity of the drug. However, in general, there may exist other factors that determine the potency of a drug. The underlying assumption for the use of the model (2) is that the inhibition, i.e. the effect of an NDNB, is proportional to the receptor occupancy by the drug. While the model (2) has been statistically tested in [4–8], the key factors affecting the validity of this assumption have not been fully discussed in literature, and it has not been examined if the assumption holds in vivo.

The purpose of this study is to explore the in vivo relationship between the inhibition and the receptor occupancy by an NDNB and to draw implications on how in vitro experimental results can be used to discuss the in vivo pharmacologic properties of NDNBs. An ordinary differential equation model based on [5, 13–15] is employed to simulate physiologic processes of the activation of receptors by acetylcholine (ACh) as well as inhibition by an NDNB. Based on the model, we examine the conditions under which the relationship between inhibition and receptor occupancy becomes nonlinear and discuss its clinically relevant implications.

## Methods

### Model of competition between acetylcholine and NDNB

**Fig. 1:**
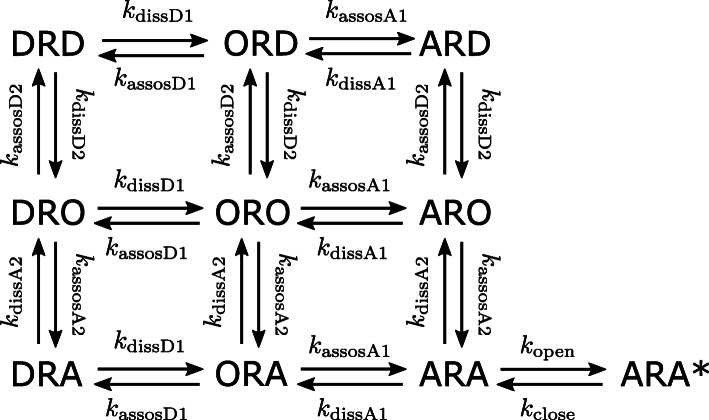
Diagram of the interactions between acetylcholine (A), NDNB (D), and the postsynaptic receptor (R). The complexes formed by binding of ACh and NDNB to AChR are represented by 3-letter symbols, where the first and last letters denote the first and second ligands occupying the sites 1 and 2, respectively. The state ARA* represents the activated AChR

At the neuromuscular junction, electrical impulses from motor nerve cause the release of transmitter, ACh, to the synaptic cleft. Then, part of the released ACh molecules bind to AChRs on the muscle membrane and results in a change in the conductance of the membrane due to the channel opening. This change causes movement of ions into the muscle cell that produces an action potential spreading over the surfaces of skeletal muscle fibers and causing muscle contraction. NDNBs act by competing with ACh for binding to the two sites of an AChR and preventing changes in the membrane conductance. To describe the competition between ACh and NDNB molecules for binding to AChRs, this paper uses the model developed in [15] with a modification to incorporate the dynamics of channel opening as described in [5, 13, 14]. The complexes formed by binding of ACh, denoted by A, and NDNB, by D, to AChR, by R, are represented by 3-letter symbols as shown in Fig. 1. The first and last letters denote the first and second ligands occupying the sites 1 and 2, respectively, and the middle letter represents the receptor R. Unoccupied sites are denoted by O, and ORO stands for free AChR. The kinetics of ACh and NDNB are represented using association rate constants *k*_assocA*i*_ and *k*_assocD*i*_, for site *#i*(*i*=1, 2), respectively. Similarly, *k*_dissA*i*_ and *k*_dissD*i*_ stand for the dissociation rate constants for ACh and NDNB. The symbol ARA stands for AChRs bound with two ACh molecules but in the closed state, and the symbol ARA* for AChRs in the open state and thus activated due to the conformational change of AChRs. The rate constants of opening and closing of AChRs are represented by *k*_open_ and *k*_close_, respectively. Then, the concentrations of these complexes can be calculated as a function of time by solving the following ordinary differential equations:

3a $$\begin{array}{*{20}l} & \frac{\mathrm{d}}{\mathrm{d} t}\mathrm{[\!A]} = - {k_{\text{decay}}} \mathrm{[\!A]} \\ &\hspace{13mm} + k_{\mathrm{dissA1}} (\mathrm{[\!ARA]} + \mathrm{[\!ARD]} + \mathrm{[\!ARO]}) \\ &\hspace{13mm} - k_{\mathrm{assocA1}} \mathrm{[\!A]} (\mathrm{[\!ORA]} + \mathrm{[\!ORD]} + \mathrm{[\!ORO]}) \\ &\hspace{13mm} + k_{\mathrm{dissA2}} (\mathrm{[\!ARA]} + \mathrm{[\!DRA]} + \mathrm{[\!ORA]}) \\ &\hspace{13mm} - k_{\mathrm{assocA2}} \mathrm{[\!A]} (\mathrm{[\!ARO]} + \mathrm{[\!DRO]} + \mathrm{[\!ORO]}), \end{array} $$

3b $$\begin{array}{*{20}l}  & { \frac{\mathrm{d}}{\mathrm{d} t}\mathrm{[\!ARA^{\ast}]} = - k_{\text{close}}\ \mathrm{[\!ARA^{\ast}]} + k_{\text{open}} \mathrm{[\!ARA]},} \end{array} $$

3c $$\begin{array}{*{20}l} & \frac{\mathrm{d}}{\mathrm{d} t}\mathrm{[\!ARA]} = k_{\mathrm{assocA1}} \mathrm{[\!ORA][A]} - k_{\mathrm{dissA1}}\mathrm{[\!ARA]} \\ &\hspace{17mm} + k_{\mathrm{assocA2}} \mathrm{[\!ARO][A]} - k_{\mathrm{dissA2}}\mathrm{[\!ARA]} \\ &\hspace{18mm} {+ k_{\text{close}} \mathrm{[\!ARA^{\ast}]} - k_{\text{open}} \mathrm{[\!ARA]},} \end{array} $$

3d $$\begin{array}{*{20}l} & \frac{\mathrm{d}}{\mathrm{d} t}\mathrm{[\!DRD]} = k_{\mathrm{assocD1}} \mathrm{[\!ORD][D]} - k_{\mathrm{dissD1}}\mathrm{[\!DRD]} \\ &\hspace{17mm} + k_{\mathrm{assocD2}} \mathrm{[\!DRO][D]} - k_{\mathrm{dissD2}}\mathrm{[\!DRD]}, \end{array} $$

3e $$\begin{array}{*{20}l} & \frac{\mathrm{d}}{\mathrm{d} t}\mathrm{[\!ARD]} = k_{\mathrm{assocA1}} \mathrm{[\!ORD][A]} - k_{\mathrm{dissA1}}\mathrm{[\!ARD]} \\ &\hspace{17mm} + k_{\mathrm{assocD2}} \mathrm{[\!ARO][D]} - k_{\mathrm{dissD2}}\mathrm{[\!ARD]}, \end{array} $$

3f $$\begin{array}{*{20}l} & \frac{\mathrm{d}}{\mathrm{d} t}\mathrm{[\!DRA]} = k_{\mathrm{assocD1}} \mathrm{[\!ORA][D]} - k_{\mathrm{dissD1}}\mathrm{[\!DRA]} \\ & \hspace{17mm} + k_{\mathrm{assocA2}} \mathrm{[\!DRO][A]} - k_{\mathrm{dissA2}}\mathrm{[\!DRA]}, \end{array} $$

3g $$\begin{array}{*{20}l} & \frac{\mathrm{d}}{\mathrm{d} t}\mathrm{[\!ARO]} = k_{\mathrm{assocA1}} \mathrm{[\!ORO][A]} - k_{\mathrm{dissA1}}\mathrm{[\!ARO]} \\ &\hspace{17mm} + k_{\mathrm{dissA2}} \mathrm{[\!ARA]} - k_{\mathrm{assocA2}} \mathrm{[\!ARO][A]} \\ &\hspace{17mm} + k_{\mathrm{dissD2}} \mathrm{[\!ARD]} - k_{\mathrm{assocD2}} \mathrm{[\!ARO] [D]}, \end{array} $$

3h $$\begin{array}{*{20}l} & \frac{\mathrm{d}}{\mathrm{d} t}\mathrm{[\!ORA]} = k_{\mathrm{assocA2}} \mathrm{[\!ORO][A]} - k_{\mathrm{dissA2}}\mathrm{[\!ORA]} \\ &\hspace{17mm} + k_{\mathrm{dissA1}} \mathrm{[\!ARA]} - k_{\mathrm{assocA1}} \mathrm{[\!ORA][A]} \\ &\hspace{17mm} + k_{\mathrm{dissD1}} \mathrm{[\!DRA]} - k_{\mathrm{assocD1}} \mathrm{[\!ORA] [D]}, \end{array} $$

3i $$\begin{array}{*{20}l} & \frac{\mathrm{d}}{\mathrm{d} t}\mathrm{[\!DRO]} = k_{\mathrm{assocD1}} \mathrm{[\!ORO][D]} - k_{\mathrm{dissD1}}\mathrm{[\!DRO]} \\ & \hspace{17mm} + k_{\mathrm{dissD2}} \mathrm{[\!DRD]} - k_{\mathrm{assocD2}} \mathrm{[\!DRO] [D]} \\ &\hspace{17mm} + k_{\mathrm{dissA2}} \mathrm{[\!DRA]} - k_{\mathrm{assocA2}} \mathrm{[\!DRO][A]}, \end{array} $$

3j $$\begin{array}{*{20}l} & \frac{\mathrm{d}}{\mathrm{d} t}\mathrm{[\!ORD]} = k_{\mathrm{assocD2}} \mathrm{[\!ORO][D]} - k_{\mathrm{dissD2}}\mathrm{[\!ORD]} \\ &\hspace{17mm} + k_{\mathrm{dissD1}} \mathrm{[\!DRD]} - k_{\mathrm{assocD1}} \mathrm{[\!ORD] [D]} \\ &\hspace{17mm} + k_{\mathrm{dissA1}} \mathrm{[\!ARD]} - k_{\mathrm{assocA1}} \mathrm{[\!ORD][A]}, \end{array} $$

where [ D] stands for the concentration of NDNB at the effect site, and *k*_decay_ for the rate constant of the decay of free ACh due to hydrolysis of ACh by acetylcholinesterase and diffusion of ACh away from the synaptic cleft. The concentration [ ORO] of the unoccupied AChR is given by

4 $$\begin{array}{*{20}l} \mathrm{[\!ORO]} =& \mathrm{[\!R]}_{\text{total}} {- {[\text{ARA}^{\ast}]}} - \mathrm{[\!ARA]} - \mathrm{[\!DRD]} \\&- \mathrm{[\!ARD]} - \mathrm{[\!DRA]}  \\ & - \mathrm{[\!ARO]} - \mathrm{[\!ORA]} - \mathrm{[\!DRO]} - \mathrm{[\!ORD]}, \end{array} $$

where [ R]_total_ stands for the concentration of the post-junctional AChRs in the synaptic cleft.

Here we provide an example of simulation results of the model (3). The setting of the parameters is shown in Table 1. Following [15], the concentration [ R]_total_ of AChRs was calculated using the number of AChRs (2.1×10^7^) at the end plates of human deltoid muscle reported in [16] and the volume of the synaptic cleft (4.5×10^13^L) of rat diaphragm reported in [17]. The initial concentration [ A]_init_ of the free ACh was calculated by assuming that the number of ACh molecules released is one tenth of the number of AChRs as discussed in [18, 19]. This means that the number of ACh molecules released is 2.1×10^6^ corresponding to the release of 300 vesicles [20] with 7000 ACh molecules in each vesicle. Thus, while the concentration of ACh itself is sufficiently higher than needed for neuromuscular transmission, due to the considerable excess of AChRs, only a small fractional amount of AChRs will be activated during neuromuscular transmission. The rate constant *k*_decay_ was determined such that the half-life of free ACh becomes 58*μ*s as calculated in [15]. The dissociation and association rate constants for ACh were tentatively set to the values obtained in [13, 14] from experiments using mouse adult AChRs. Similarly, the rate constants for NDNBs were set to tentative values based on experiments in [4, 5] using mouse embryonic and adult AChRs. Since the values of these rate constants may be different between human and mouse AChRs, these constants will be varied in a systematic way.

**Table 1:** Setting of parameters for numerical simulation in base case

Symbol	Meaning	Value
[ R]_total_	Concentration of AChRs in the synaptic cleft	7.75×10^−5^M [15]
[ A]_init_	Initial concentration of ACh immediately after the stimulus	7.75×10^−6^M [15]
*k*_decay_	Rate constant of the decay of the concentration of free ACh	1.2×10^4^s^−1^ [15]
*k*_dissA1_	Dissociation rate constant for ACh with site1 of AChR	1.8×10^4^s^−1^ [13, 14]
*k*_dissA2_	Dissociation rate constant for ACh with site2 of AChR	1.8×10^4^s^−1^ [13, 14]
*k*_assocA1_	Association rate constant for ACh with site1 of AChR ^∗^	1.1×10^8^M^−1^.*s*^−1^ [13, 14]
*k*_assocA2_	Association rate constant for ACh with site2 of AChR ^∗^	1.1×10^8^M^−1^s^−1^ [13, 14]
*k*_dissD1_	Dissociation rate constant for NDNB with site1 of AChR ^∗^	10s^−1^ [4, 5]
*k*_dissD2_	Dissociation rate constant for NDNB with site2 of AChR ^∗^	10s^−1^ [4, 5]
*k*_assocD1_	Association rate constant for NDNB with site1 of AChR ^∗^	1.0×10^8^M^−1^s^−1^ [4, 5]
*k*_assocD2_	Association rate constant for NDNB with site2 of AChR ^∗^	1.0×10^8^M^−1^s^−1^ [4, 5]
*k*_close_	Rate constant of channel closing of AChR	1.2×10^3^s^−1^ [14]
*k*_open_	Rate constant of channel opening of AChR	5.0×10^4^s^−1^ [14]

∗ These dissociation and association rate constants are varied in numerical analysis

**Fig. 2:**
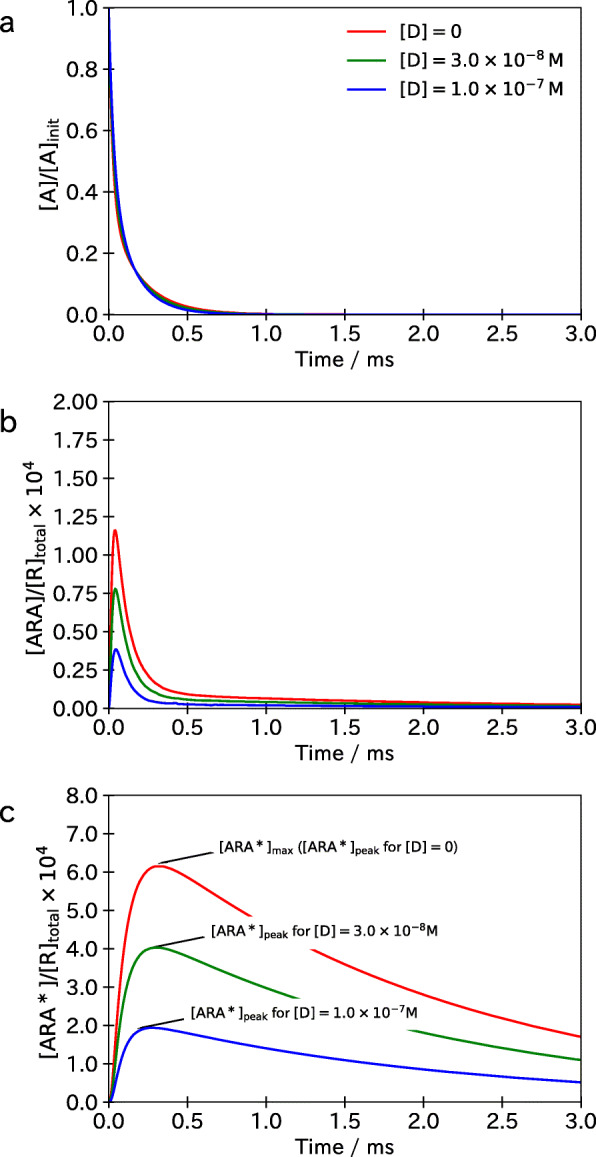
An example of simulation results of the model (3). **a** The time course of the concentration [ A] of free ACh. **b** The time course of the concentration [ ARA] of the complex ARA representing the activated AChRs

Figure 2 shows the time courses of the concentrations of free ACh, [ A], of diliganded AChRs at the closed state, [ ARA], and of the activated AChRs, [ARA^∗^], after the arrival of an electrical impulse from motor nerve and the release of ACh at *t*=0. The initial concentrations for the nine complexes at *t*=0 were defined by the steady state concentrations before the release of ACh. The concentration of the NDNB was set to one of [ D]=0,3.0×10^−8^M, and 1.0×10^−7^M. As shown in the upper panel of Fig. 2, the concentration of ACh rapidly decreases due to the hydrolysis of ACh by acetylcholinesterase and binding of ACh to AChRs. The time course of [ ARA] is shown in the middle panel of Fig. 2. The concentration [ ARA] raises rapidly and decays after reaching the peak at around *t*=0.04 ms. Subsequently, as shown in the lower panel, the concentration [ARA^∗^] raises and reaches its peak at around *t*=0.3 ms. The highest [ARA^∗^], denoted by [ARA^∗^]_max_, is attained in the absence of NDNB, i.e., [ D]=0, and the peak concentration of the activated AChRs [ARA^∗^]_peak_ decreases with the increase in the concentration of [ D].

With this model, the effect of an NDNB can be quantified by a fraction of activated AChRs given by [ARA^∗^]_peak_/[ARA^∗^]_max_. This definition corresponds to the relative current *I*_antag_/*I*_0_ used in in vitro experiments under the assumption that the membrane conductance is proportional to the number of activated AChRs, i.e. the number of opened ion channels:

5 $$\begin{array}{*{20}l}  \frac{I_{\text{antag}}}{I_{0}} = {\frac{[\text{ARA}^{\ast}]_{\text{peak}}}{[\text{ARA}^{\ast}]_{\text{max}}}}. \end{array} $$

### Theoretical analysis of the competition model

To conduct the subsequent numerical analysis in a systematic way, here we theoretically analyze the model (3). Specifically, we provide a simplified representation of the model under the assumption that the dissociation rate constants *k*_dissD1_ and *k*_dissD2_ of an NDNB are much smaller than other rate constants such as *k*_dissA1_,*k*_dissA2_ and *k*_decay_. For cisatracurium, the dissociation rate constants are reported in [5] as 13s^−1^ and 34s^−1^ for mouse adult and embryonic AChRs, respectively. Also, for (+)-tubocurarine and pancuronium, these values are reported in [4] as 2.1s^−1^ and 5.9s^−1^, respectively. Whereas, the dissociation rate constants of ACh and the rate constant of hydrolysis are estimated as 18000s^−1^ and 12000s^−1^, respectively [13, 15].

The simplified model is given in a dimensionless representation. For example, a dimensionless time *τ* and a concentration of ACh, *x*_A_, can be defined as

6 $$\begin{array}{*{20}l} \tau := t \cdot {k_{\text{decay}}}, \quad x_{\mathrm{A}} := \frac{\mathrm{[\!A]}}{ K_{\mathrm{A1}}}, \end{array} $$

where *K*_A1_ stands for the dissociation equilibrium constant for ACh with the site *#*1 given by *k*_dissA1_/*k*_assocA1_. Dimensionless variables $x^{\ast }_{\text {ARA}}$ and *x*_DRD_ for the concentrations of the complex AR*A*^∗^ and DRD are given by

7 $$\begin{array}{*{20}l} {x^{\ast}_{\text{ARA}} := \frac{{[\text{ARA}^{\ast}]}}{\mathrm{[\!R]}_{\text{total}}}}, \quad x_{\text{DRD}} := \frac{\mathrm{[\!DRD]}}{\mathrm{[\!R]}_{\text{total}} }. \end{array} $$

Similarly, the dimensionless variables *x*_ARA_,*x*_ARD_,*x*_ARD_,*x*_DRA_,*x*_ARO_,*x*_ORA_,*x*_DRO_, and *x*_ORD_ for the remaining seven complexes can be defined by dividing by [ R]_total_. By using these dimensionless variables, the simplified model is given as follows (see Appendix for its derivation):

8a $$\begin{array}{*{20}l} {}&\frac{\mathrm{d}}{\mathrm{d} \tau}x_{\mathrm{A}} = - x_{\mathrm{A}} +\kappa_{\mathrm{A1}}\lambda_{\mathrm{A1}} \{ x_{\text{ARA}} - x_{\mathrm{A}} (O_{\text{ORD}}(\delta) - x_{\text{ARD}}) \}  \\ {}& \hspace{16mm} +\kappa_{\mathrm{A2}}\lambda_{\mathrm{A2}} \{ x_{\text{ARA}} - x_{\mathrm{A}} (O_{\text{DRO}}(\delta) - x_{\text{DRA}}) \}  \\ {}& \hspace{16mm} +\kappa_{\mathrm{A1}}\lambda_{\mathrm{A1}} \{ x_{\text{ARD}} + x_{\text{ARO}} - x_{\mathrm{A}} (1-x_{\text{ARA}}-x_{\text{ARO}} - O_{\text{total}}(\delta)) \}  \\ {}& \hspace{16mm} +\kappa_{\mathrm{A2}}\lambda_{\mathrm{A2}} \{ x_{\text{DRA}}+ x_{\text{ORA}} - x_{\mathrm{A}} (1-x_{\text{ARA}} -x_{\text{ORA}}- O_{\text{total}}(\delta)) \},  \end{array} $$

8b $$\begin{array}{*{20}l} {}& {\frac{\mathrm{d}}{\mathrm{d} \tau}x^{\ast}_{\text{ARA}} = - \kappa_{\text{close}} x^{\ast}_{\text{ARA}} + \kappa_{\text{open}}x_{\text{ARA}}, } \end{array} $$

8c $$\begin{array}{*{20}l} & \frac{\mathrm{d}}{\mathrm{d} \tau}x_{\text{ARA}} = \kappa_{\mathrm{A1}} \left(x_{\text{ORA}} x_{\mathrm{A}} - x_{\text{ARA}} \right) + \kappa_{\mathrm{A2}} \left(\mu_{\mathrm{A}} x_{\text{ARO}} x_{\mathrm{A}} - x_{\text{ARA}} \right)  \\ & \hspace{16mm} {+ \kappa_{\text{close}} x^{\ast}_{\text{ARA}} - \kappa_{\text{open}}x_{\text{ARA}}}, \end{array} $$

8d $$\begin{array}{*{20}l} & \frac{\mathrm{d}}{\mathrm{d} \tau}x_{\text{ARO}} = \kappa_{\mathrm{A1}} \left\{ x_{\mathrm{A}} (1-x_{\text{ARA}}-x_{\text{ARO}}-x_{\text{ORA}}- O_{\text{total}}(\delta))- x_{\text{ARO}} \right\}  \\ & \hspace{16mm} +\kappa_{\mathrm{A2}} \left(x_{\text{ARA}} - \mu_{\mathrm{A}} x_{\text{ARO}} x_{\mathrm{A}} \right), \end{array} $$

8e $$\begin{array}{*{20}l} & \frac{\mathrm{d}}{\mathrm{d} \tau}x_{\text{ORA}} = \kappa_{\mathrm{A2}} \left\{ \mu_{\mathrm{A}} x_{\mathrm{A}} (1-x_{\text{ARA}}-x_{\text{ARO}}-x_{\text{ORA}}- O_{\text{total}}(\delta)) - x_{\text{ORA}} \right\}  \\ &\hspace{16mm} + \kappa_{\mathrm{A1}} \left(x_{\text{ARA}} - x_{\text{ORA}} x_{\mathrm{A}} \right), \end{array} $$

8f $$\begin{array}{*{20}l} & \frac{\mathrm{d}}{\mathrm{d} \tau}x_{\text{ARD}} = \kappa_{\mathrm{A1}} \left\{ x_{\mathrm{A}} (O_{\text{ORD}}(\delta) - x_{\text{ARD}}) - x_{\text{ARD}} \right\}, \end{array} $$

8g $$\begin{array}{*{20}l} & \frac{\mathrm{d}}{\mathrm{d} \tau}x_{\text{DRA}}= \kappa_{\mathrm{A2}} \left\{ \mu_{\mathrm{A}} x_{\mathrm{A}} (O_{\text{DRO}}(\delta) - x_{\text{DRA}}) - x_{\text{DRA}} \right\}, \end{array} $$

where *κ*_A1_,*κ*_A2_, *κ*_open_ and *κ*_close_ are the dimensionless parameters representing normalized rate constants given by

9 $$\begin{array}{*{20}l} & \kappa_{\mathrm{A1}} := \frac{k_{\mathrm{dissA1}} }{ {k_{\text{decay}}} }, \quad \kappa_{\mathrm{A2}} := \frac{k_{\mathrm{dissA2}} }{ {k_{\text{decay}}} },  \\ & {\kappa_{\text{open}} := \frac{k_{\text{open}} }{ {k_{\text{decay}}} }, \quad \kappa_{\text{close}} := \frac{k_{\text{close}} }{ {k_{\text{decay}}} }, } \end{array} $$

The parameters *λ*_A1_,*λ*_A2_, and *μ*_A_are the dimensionless parameters representing affinities of ACh to the binding sites of the AChR given by

10 $$\begin{array}{*{20}l} \lambda_{\mathrm{A1}} := \frac{\mathrm{[\!R]}_{\text{total}}}{K_{\mathrm{A1}} }, \quad \lambda_{\mathrm{A2}} := \frac{\mathrm{[\!R]}_{\text{total}}}{K_{\mathrm{A2}} }, \quad \mu_{\mathrm{A}} := \frac{K_{\mathrm{A1}}}{K_{\mathrm{A2}}}, \quad \end{array} $$

with *K*_A2_:=*k*_dissA2_/*k*_assocA2_, and finally, *μ*_D_ and *δ* are the dimensionless parameters representing the site-selectivity and concentration of NDNB, respectively, given by

11 $$\begin{array}{*{20}l} \mu_{\mathrm{D}} := \frac{K_{\mathrm{D1}}}{K_{\mathrm{D2}}}, \quad \delta := \frac{{[\!\mathrm{D}]}}{ K_{\mathrm{D1}} }, \end{array} $$

with *K*_D1_:=*k*_dissD1_/*k*_assocD1_ and *K*_D2_:=*k*_dissD2_/*k*_assocD2_. Furthermore, *O*_DRD_,*O*_ORD_ and *O*_DRO_ are functions of *δ* standing for the fractions of the complex DRD, ORD, and DRO before the release of ACh given by

12a $$\begin{array}{*{20}l} O_{\text{DRD}}(\delta) &:= \frac{ \mu_{\mathrm{D}} \delta^{2} }{ (1 + \delta)(1 + \mu_{\mathrm{D}} \delta) }, \end{array} $$

12b $$\begin{array}{*{20}l} O_{\text{ORD}}(\delta) &:= \frac{ \mu_{\mathrm{D}} \delta }{ (1 + \delta)(1 + \mu_{\mathrm{D}} \delta) }, \end{array} $$

12c $$\begin{array}{*{20}l} O_{\text{DRO}}(\delta) &:= \frac{ \delta }{ (1 + \delta)(1 + \mu_{\mathrm{D}} \delta)}. \end{array} $$

The total occupancy *O*_total_ of the AChRs by NDNB is given by

13a $$\begin{array}{*{20}l} O_{\text{total}}(\delta) & := O_{\text{DRD}}(\delta) + O_{\text{ORD}}(\delta) + O_{\text{DRO}}(\delta). \end{array} $$

**Fig. 3:**
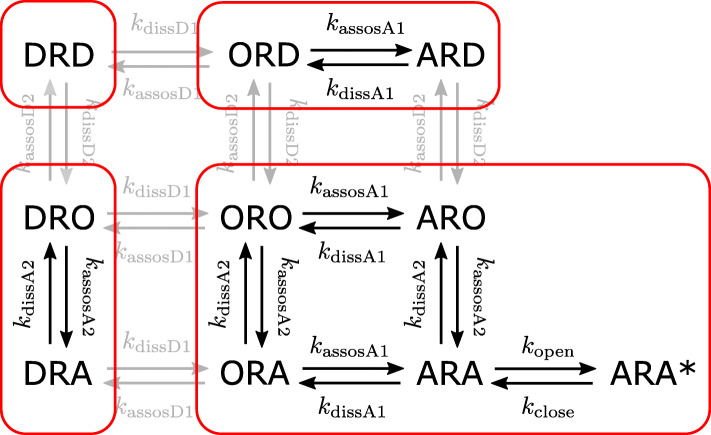
Diagram of the interactions between acetylcholine (A), NDNB (D), and the postsynaptic receptor (R) based on the simplified model (8). The state DRD and the pairs of states (ORD, ARD) and (DRO, DRA) can be viewed as separated from the states describing the activation of AChRs, while interaction between these states may occur through the dynamics of free ACh given by (8a)

From the simplified model (8), several insights can be obtained on the relationship between the receptor occupancy by NDNBs and the dynamics of activation of AChRs. First, it can be seen that the model (8) has only one parameter *μ*_D_ characterizing NDNBs. Thus, the properties of NDNBs are completely determined by the parameter *μ*_D_ representing the site-selectivity of NDNBs. This fact implies that even in the simulations of the original model (3), the results will be almost unchanged if the parameter *μ*_D_ kept constant and the parameters *κ*_D1_:=*k*_dissD1_/*k*_decay_ and *κ*_D2_:=*k*_dissD2_/*k*_decay_ are small enough to validate the simplification (see Appendix for details of the validity of the simplification). This observation facilitates the numerical analysis of the original model (3) by varying the parameters of the model in a systematic way as demonstrated in the rest of the paper. Second, from the model Eq. 8, it can be seen that the entire states during competition can be viewed as divided into 4 parts as shown in Fig. 3. Note that owing to an appropriate derivation of the dimensionless representation of the model, not only the terms for dissociation of NDNBs but also association terms are eliminated from the original model. As a result, the state DRD and the pairs of states (ORD, ARD) and (DRO, DRA) can be viewed as separated from the states describing the activation of AChRs, while interaction between these states may occur through the dynamics of free ACh given by (8a). Specifically, although the fraction of the state DRD will not change during the competition, the fractions of ORD and DRO will change due to association and dissociation with ACh molecules. Therefore, even if the total occupancy *O*_total_ is the same, the fraction of the activated AChRs will decrease with the increase in the occupancies *O*_ORD_ and *O*_DRO_ because free ACh will decrease due to association with ORD and DRO. Note that the ratio of ORD and DRO to *O*_total_ is determined by the parameter *μ*_D_. Finally, the difference between the receptor binding model (2) and the competition-based models (3) and (8) can be clarified. By using the notation introduced above, the model (2) can be rewritten as follows:

14 $$\begin{array}{*{20}l} \frac{I_{\text{antag}}}{I_{0}} = \frac{1}{1 + \delta + \mu_{\mathrm{D}} \delta + \mu_{\mathrm{D}} \delta^{2}} = 1 - O_{\text{total}}(\delta) \end{array} $$

Thus, the model (2) is clearly based on the assumption that the inhibition is proportional to the total occupancy *O*_total_, while the models (3) and (8) describe the effect of the partial occupancies *O*_ORD_ and *O*_DRO_ on the activation of AChRs during competition.

### Method of numerical analysis on the relationship between inhibition and receptor occupancy

The relationship between the inhibition and the receptor occupancy by an NDNB is studied via numerical simulations of the original model (3). To visualize the results, the fraction of activated AChRs given by [ARA^∗^]_peak_/[ARA^∗^]_max_ is calculated under various concentrations of the NDNB (100 values between [ D]=1.0×10^−14^M and 1.0×10^−5^M that are spaced equidistantly on a logarithmic scale, and [ D]=0) and plotted versus the total occupancy *O*_total_. For calculating the peak concentration of ARA, the ordinary differential equation model (3) is numerically solved using the Fortran solver LSODA provided by the python package SciPy (Version 1.5.2).

The parameters of the model (3) are varied in a systematic way based on information provided by literature and the findings of the theoretical analysis as explained in the following. First, we investigate the effect of varying the kinetic constants for ACh and NDNB. For ACh, the values of *k*_dissA1_,*k*_dissA2_,*k*_assocA1_, and *k*_assocA2_ presented in Table 1 were determined by experiments using mouse adult AChRs. However, it is known that the EC_50_, the concentration of ACh where the half of the AChRs are activated, for human adult AChRs is smaller than that for mouse adult AChRs: EC_50_=8.48×10^−6^M or 7.91×10^−6^M for human adult AChRs [10] and EC_50_=1.6×10^−5^M for mouse adult AChRs [13]. Since this implies that the affinity of ACh is different between human and mouse AChRs, we investigated the effect of varying the constants *K*_A1_ and *K*_A2_ by changing *k*_assocA1_ and *k*_assocA2_ while *k*_dissA1_ and *k*_dissA2_ kept constant. Furthermore, since it has been reported in [13] that the two binding sites of mouse adult AChR have similar affinities, we assume that it is also the case for human adult AChR and the parameters *K*_A1_ and *K*_A2_ are equal. Thus, we investigate the effect of varying the values of *K*_A1_=*K*_A2_=*K*_A_, which corresponds to *κ*_A1_=*κ*_A2_ and *μ*_A_=1. For the kinetic constants of an NDNB, the effect of varying the parameter *μ*_D_ is investigated, since this parameter completely characterizes the properties of the NDNB as far as the dissociation constants *k*_dissD1_ and *k*_dissD2_ are small enough. The value of parameter *μ*_D_ was assigned by changing the value of *k*_assocD2_, while *k*_dissD1_,*k*_dissD2_ and *k*_assocD1_ kept constant at the values shown in Table 1. Also, the values of the dissociation constants *k*_dissD1_ and *k*_dissD2_ are varied to explore the difference between the original model (3) and the reduced-order model (8).

Furthermore, we examine the effect of varying the initial concentration [ A]_init_ representing the concentration of ACh immediately after the release of ACh at *t*=0. It has been known that the number of ACh molecules released in vivo is one tenth of the number of AChRs at the synaptic cleft [15]. In this paper, following [15], the concentrations of AChRs and the initial ACh were set as [ R]_total_=7.75×10^−5^M and [ A]_init_=7.75×10^−6^M, respectively. Under this setting, only a small fraction (at least less than one tenth) of the total receptor population will be activated in vivo. However, in many in vitro experiments, a quite high concentration of ACh is used [4–8] such that nearly 50*%* (nearly EC_50_) of the AChRs are activated or more than 90*%* of AChRs are activated. Thus, we investigated the effect of varying [ A]_init_ in a wide range of concentrations from [ A]_init_=0.0562×[ R]_total_ to 5.62×[ R]_total_.

## Results

**Fig. 4:**
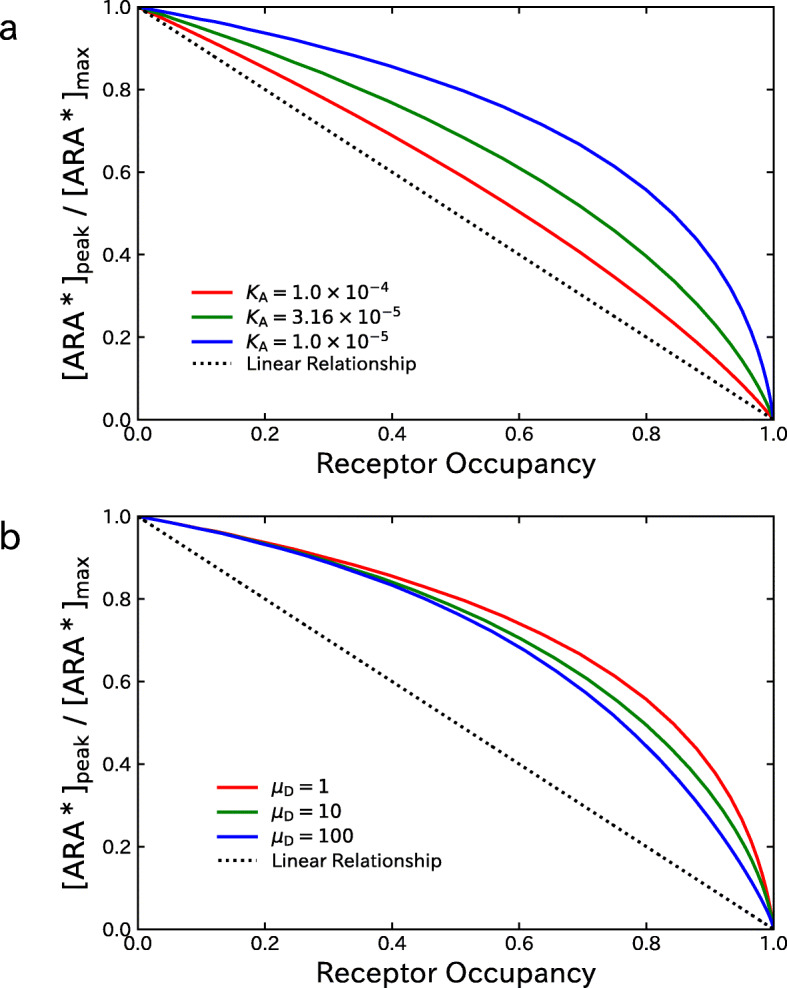
Effect of varying kinetic constants for ACh and NDNB on the relationship between the fraction of activated AChRs, [ARA^∗^]_peak_/[ARA^∗^]_max_, and the receptor occupancy *O*_total_. **a** Results of varying the equilibrium dissociation constants for ACh under *K*_A_=*K*_A1_=*K*_A2_ (*κ*_A1_=*κ*_A2_ and *μ*_A_=1). **b** Results of varying the site-selectivity *μ*_D_=*K*_D1_/*K*_D2_ of an NDNB

The upper panel of Fig. 4 shows the relationship between the fraction of activated AChRs given by [ARA^∗^]_peak_/[ARA^∗^]_max_ and the receptor occupancy *O*_total_ evaluated under various settings of the dissociation equilibrium constants for ACh. The values of *K*_A1_=*K*_A2_=*K*_A_ is one of 1.0×10^−4^M,3.16×10^−5^M and 1.0×10^−5^M, and shown by *red*, *green* and *blue* lines, respectively. The dotted line in the figure shows the linear relationship corresponding to the model (2). The results clearly show that there are nonlinear relationships between [ARA^∗^]_peak_/[ARA^∗^]_max_ and *O*_total_ in all the cases. Furthermore, it can be confirmed that the extent of nonlinearity increases with the decrease in the equilibrium constant *K*_A_. In the subsequent analyses, to highlight the effects of varying other parameters, we assigned the value of 1.0×10^−5^M to *K*_A_, where the extent of nonlinearity was most prominent. Then, the lower panel of Fig. 4 shows the results under various settings of *μ*_D_ representing the site-selectivity of an NDNB. The *red*, *green* and *blue* lines show the results for *μ*_D_=1, 10, and 100, respectively. It can be seen that the relationships between [ARA^∗^]_peak_/[ARA^∗^]_max_ and *O*_total_ are nonlinear in all the cases, and the extent of nonlinearity decreases with the increase in *μ*_D_.

**Fig. 5:**
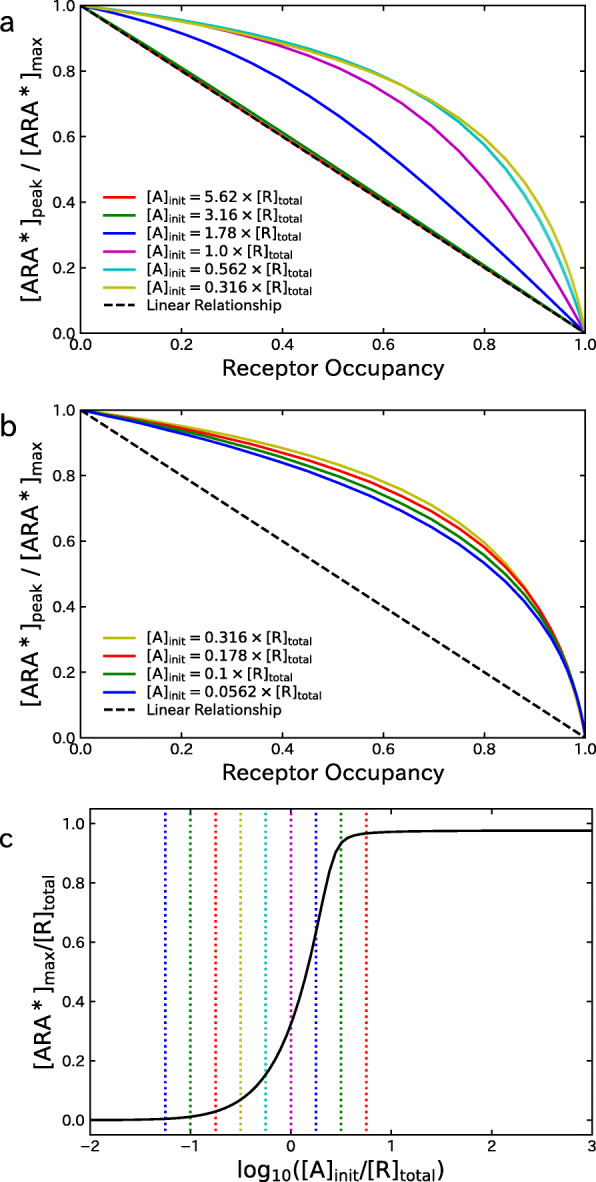
Effect of varying the initial concentration [ A]_init_ of ACh on the numerical results. **a** The relationship between the fraction of activated AChRs, [ARA^∗^]_peak_/[ARA^∗^]_max_, and the receptor occupancy *O*_total_ under various settings of [ A]_init_. **b** The concentration-effect relationship between the activated AChRs in the absence of NDNBs and the initial concentration [ A]_init_

Next, the upper and the middle panels of Fig. 5 show the relationship between the fraction of activated AChRs, [ARA^∗^]_peak_/[ARA^∗^]_max_, and the receptor occupancy *O*_total_ under various settings of the initial concentration of ACh, [A]_init_. In the upper panel, the initial concentration [A]_init_ is decreased from 5.62×[R]_total_ to 0.316×[R]_total_, and it can be seen that the extent of nonlinearity increases with the decrease in [A]_init_. Also, in the middle panel, the initial concentration is further decreased to 0.0562×[R]_total_. The extent of nonlinearlity slightly decreases in this range of parameter setting, and thus the nonlinearity is most prominent at [ A]_init_=0.316×[R]_total_. Interestingly, the relationship between [ARA^∗^]_peak_/[ARA^∗^]_max_ and *O*_total_ becomes almost linear when the value of [A]_init_ is larger than 3.16×[R]_total_. To clarify the meaning of this result, the lower panel of Fig. 5 shows the concentration-response relationship between the concentration [A]_init_ and the activated AChRs [ARA^∗^]_max_. Note that the [ARA^∗^]_max_ is defined for each setting of [ A]_init_ as the highest [ARA^∗^] under various settings of the concentration of an NDNB, and it is attained in the absence of NDNB. At the setting of [ A]_init_=0.1×[R]_total_, which corresponds to the in vivo situation [15], only a fraction of AChRs are activated even in the absence of NDNBs, and result in a highly nonlinear relationship between [ARA^∗^]_peak_/[ARA^∗^]_max_ and *O*_total_. However, at the setting of [ A]_init_≥3.16×[R]_total_, more than 92*%* of AChRs are activated in the absence of NDNB, and in this case, the relationship between [ARA^∗^]_peak_/[ARA^∗^]_max_ and *O*_total_ becomes linear.

**Fig. 6:**
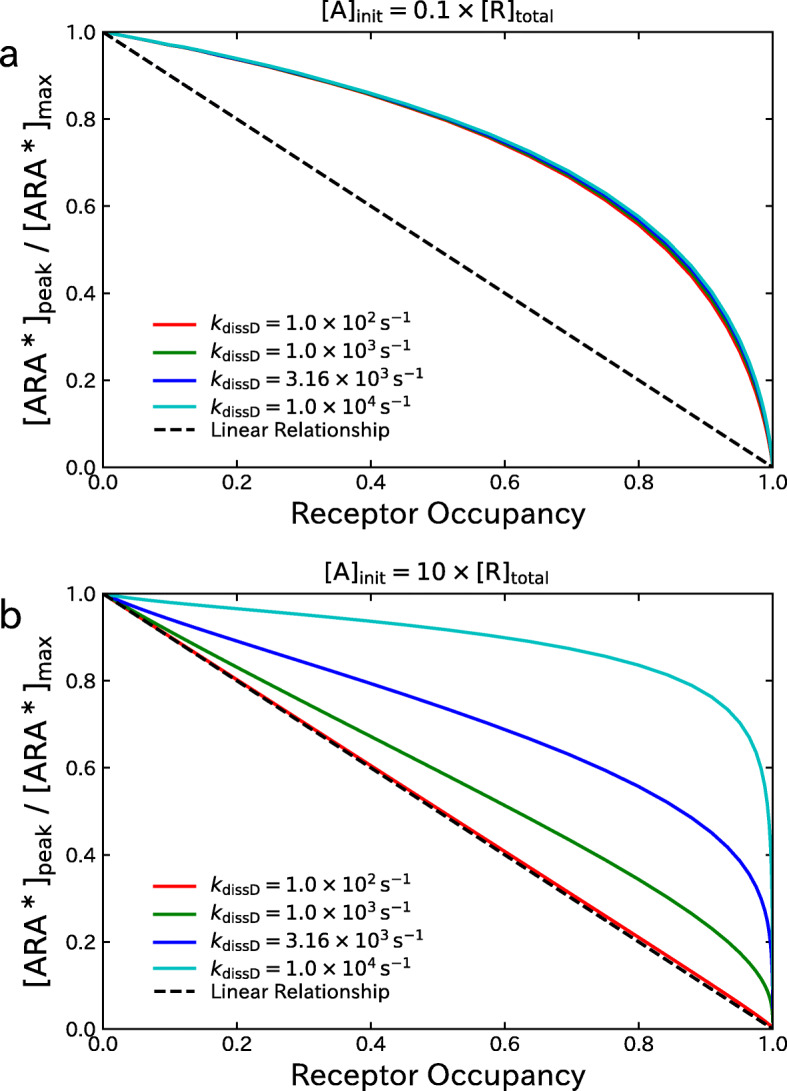
Effect of varying the dissociation rate constants *k*_dissD1_ and *k*_kdissD2_ on the relationship between the fraction of activated AChRs, [ARA^∗^]_peak_/[ARA^∗^]_max_, and the receptor occupancy *O*_total_. **a** Results under the setting of [ A]_init_=0.1×[R]_total_ corresponding to in vivo concentration. **b** Results under the setting of [ A]_init_=10×[R]_total_ corresponding to in vitro concentration

Finally, the effect of varying the dissociation rate constants *k*_dissD1_ and *k*_dissD2_ of NDNB was examined under the setting of identical rate constants, i.e. *k*_dissD1_=*k*_dissD2_=*k*_dissD_. The upper panel of Fig. 6 shows the relationship between the fraction of activated AChRs, [ARA^∗^]_peak_/[ARA^∗^]_max_, and the receptor occupancy *O*_total_ under the setting of [ A]_init_=0.1×[R]_total_ corresponding to in vivo concentration. With this setting, the results are almost unchanged even when the rate constant *k*_dissD_ is as large as *k*_dissA1_,*k*_dissA2_, and *k*_decay_. This implies that the model simplification performed in this paper can be validated for a wide range of settings of *k*_dissD_. The lower panel of Fig. 6 shows the results under the setting of [ A]_init_=10×[R]_total_ corresponding to in vitro concentration. In this case, it can be seen that the results are highly affected by the setting of the dissociation rate constant *k*_dissD_. Furthermore, the relationships between [ARA^∗^]_peak_/[ARA^∗^]_max_ and *O*_total_ are no longer linear when the rate constant *k*_dissD_ becomes large even if the concentration [ A]_init_ is large. This implies that the model simplification can be validated only for small values of the parameter *k*_dissD_.

## Discussion

We theoretically and numerically investigated the relationship between inhibition and receptor occupancy by NDNBs. While the two-site receptor binding model (2), which assumes a linear relationship between the inhibition and the receptor occupancy by an NDNB, has been statistically tested in [4–8] for several in vitro experimental settings, it has not been studied in literature under which conditions the above assumption holds nor if the assumption remains valid in vivo. To consider these problems, an ordinary differential equation model was introduced based on [5, 13–15] to simulate the physiologic processes of activation of receptors by ACh as well as inhibition by an NDNB. The theoretical analysis performed in this paper clarified that under the assumption that the dissociation rate constants for an NDNB are small and with an appropriate nondimensionalization, the characteristics of an NDNB can be completely determined by a single parameter *μ*_D_ representing the site-selectivity of the NDNB for two binding sites of AChRs. We then performed numerical analysis of the model by plotting the fractional amounts of the activated AChRs as a function of the receptor occupancy. The numerical results show that under a setting of parameters reflecting in vivo environment, there is a nonlinear relationship between the inhibition and the receptor occupancy, indicating limitation of the applicability of the receptor binding model. Furthermore, it has been shown that the extent of nonlinearity depends on the parameters representing kinetic constants for ACh or NDNBs and the concentration of ACh.

Regarding the nonlinear relationship between the effect and the receptor occupancy by an NDNB, it has been well known that the twitch strength (the degree of muscle contraction) observed in vivo is not proportional to the receptor occupancy due to the high margin of safety [21]. The origin of the safety margin is a copious density of AChRs on the post-synaptic membrane and the fact that only a small fraction of AChRs needs to be activated to trigger the occurrence of an action potential and the contraction of the associated muscle fiber. Thus, the nonlinearity due to the safety margin means that the response of muscle fiber is not proportional to the fraction of activated AChRs, and the extent of nonlinearity would not be affected by the properties of an NDNB. However, this paper focused on the nonlinearity in the relationship between the receptor occupancy and the fraction of activated AChRs, and the extent of nonlinearity is affected by the properties of an NDNB. In particular, it has been revealed in this paper that the effect of an NDNB on the extent of nonlinearity can be characterized by a single parameter *μ*_D_ representing the site-selectivity of the NDNB.

Although the model used and simulations performed in this paper are intended to describe in vivo situations, our finding that the extent of nonlinearity is affected by the concentration of ACh is consistent with results observed through in vitro experiments. In [9, 10], it was found that the IC_50_ for several clinically used NDNBs, cis-atracrium, rocuronium, and vecronium, decreased with the increase in the concentration of ACh. With these results, it was concluded in [9, 10, 22] that this phenomenon indicates a noncompetitive component of inhibition under the idea that the enhancement of the inhibition was caused by a mechanism different from competitive one occurred by NDNBs in combination with high concentration of ACh. However, such a shift in the values of IC_50_ can also be explained by a change in the relationship between the receptor occupancy and the fraction of activated AChRs. As demonstrated in the upper panel of Fig. 5, the receptor occupancy *O*_total_ at which [ARA^∗^]_peak_/[ARA^∗^]_max_ (which corresponds to the relative current) takes the value of 0.5 increases with the decrease in the concentration of ACh, meaning that the IC_50_ increases with the decrease in the concentration of ACh. This shift in the IC_50_ is consistent with the observation in [9, 10] and occurs under a totally competitive mechanism of inhibition by an NDNB.

Interestingly, it was found that the relationship between the fraction of activated AChRs and the receptor occupancy became linear when the concentration of ACh was sufficiently large and the dissociation rate constants of an NDNB were sufficiently small. This finding may provide a reasonable justification of the use of the two-site model (2) in the analysis of kinetic constants for NDNBs through in vitro experiments. At least, the condition of large concentration of ACh is satisfied in the experiments reported in [4–8], where concentrations that opens about 93*%* to 95*%* of the AChRs were used. However, further consideration is needed to identify the conditions needed for the justification of the model (2), because the present study did not take into account the effect of desensitization of AChRs, which is the main cause of the decay in a measured current observed during in vitro experiments.

## Conclusion

The relationship between the inhibition and the receptor occupancy by an NDNB was theoretically and numerically investigated. While a receptor binding model, which assumes a linear relationship, may be effective for estimating affinity of an NDNB through in vitro experiments, the model do not directly describe in vivo pharmacologic properties of NDNBs, because the nonlinearity between the inhibition and the receptor occupancy causes the modulation of the resultant concentration-effect relationships of NDNBs. It was found that the effect of characteristics of an NDNB on the extent of nonlinearity can be identified by a single parameter representing the site-selectivity of an NDNB.

## Appendix

This appendix provides a detailed derivation of the simplified model (8). Specifically, we derive a dimensionless representation of the original model based on the technique of scaling [23] and perform model-order reduction based on singular perturbation theory [24, 25]. The scaling of the model (3) can be done by introducing dimensionless variables. For example, a dimensionless time *τ* and a concentration of ACh, *x*_A_, can be defined as

15 $$\begin{array}{*{20}l} \tau := t \cdot {k_{\text{decay}} }, \quad x_{\mathrm{A}} := \frac{\mathrm{[\!A]}}{ K_{\mathrm{A1}} }, \end{array} $$

where *K*_A1_ stands for the dissociation equilibrium constant for ACh with the site *#*1 given by *k*_dissA1_/*k*_assocA1_. Dimensionless variables $x^{\ast }_{\text {ARA}}$ and *x*_DRD_ for the concentrations of the complex AR*A*^∗^ and DRD are given by

16 $$\begin{array}{*{20}l} {x^{\ast}_{\text{ARA}} := \frac{{[\text{ARA}^{\ast}]} }{ \mathrm{[\!R]}_{\text{total}} }}, \quad x_{\text{DRD}} := \frac{\mathrm{[\!DRD]} }{ \mathrm{[\!R]}_{\text{total}} }. \end{array} $$

Similarly, the dimensionless variables *x*_ARA_, *x*_ARD_,*x*_ARD_,*x*_DRA_,*x*_ARO_,*x*_ORA_,*x*_DRO_, and *x*_ORD_ for the remaining seven complexes can be defined by dividing by [ R]_total_. By substituting these dimensionless variables to the model (3), the following equations can be derived:

17a $$\begin{array}{*{20}l} {}& \frac{\mathrm{d}}{\mathrm{d} \tau} x_{\mathrm{A}} = - x_{\mathrm{A}} + \kappa_{\mathrm{A1}} \lambda_{\mathrm{A1}} \{ x_{\text{ARA}} + x_{\text{ARD}} + x_{\text{ARO}}  \\ {}& \hspace{13mm} - x_{\mathrm{A}} (x_{\text{ORA}} + x_{\text{ORD}} +x_{\text{ORO}}) \}  \\ {}&\hspace{13mm} + \kappa_{\mathrm{A2}} \{\lambda_{\mathrm{A1}}(x_{\text{ARA}} + x_{\text{DRA}}+ x_{\text{ORA}})  \\ {}&\hspace{13mm} - \lambda_{\mathrm{A2}} x_{\mathrm{A}} (x_{\text{ARO}} + x_{\text{DRO}}+ x_{\text{ORO}}) \}, \end{array} $$

17b $$\begin{array}{*{20}l} {}& {\frac{\mathrm{d}}{\mathrm{d} \tau}x^{\ast}_{\text{ARA}} = - \kappa_{\text{close}} x^{\ast}_{\text{ARA}} + \kappa_{\text{open}}x_{\text{ARA}},} \end{array} $$

17c $$\begin{array}{*{20}l} {}& \frac{\mathrm{d}}{\mathrm{d} \tau}x_{\text{ARA}} = \kappa_{\mathrm{A1}} \left(x_{\text{ORA}} x_{\mathrm{A}} - x_{\text{ARA}} \right) + \kappa_{\mathrm{A2}} \left(\mu_{\mathrm{A}} x_{\text{ARO}} x_{\mathrm{A}} - x_{\text{ARA}} \right)  \\ {}&\hspace{16mm} {+ \kappa_{\text{close}} x^{\ast}_{\text{ARA}} - \kappa_{\text{open}}x_{\text{ARA}},} \end{array} $$

17d $$\begin{array}{*{20}l} {}& \frac{\mathrm{d}}{\mathrm{d} \tau}x_{\text{DRD}} = \kappa_{\mathrm{D1}} \left(x_{\text{ORD}} \delta - x_{\text{DRD}} \right) + \kappa_{\mathrm{D2}} \left(\mu_{\mathrm{D}}x_{\text{ORD}} \delta - x_{\text{DRD}} \right),  \end{array} $$

17e $$\begin{array}{*{20}l} {}& \frac{\mathrm{d}}{\mathrm{d} \tau}x_{\text{ARD}} = \kappa_{\mathrm{A1}} \left(x_{\text{ORD}} x_{\mathrm{A}} - x_{\text{ARD}} \right) + \kappa_{\mathrm{D2}} \left(\mu_{\mathrm{D}} x_{\text{ARO}} \delta - x_{\text{ARD}} \right),  \end{array} $$

17f $$\begin{array}{*{20}l} {}& \frac{\mathrm{d}}{\mathrm{d} \tau}x_{\text{DRA}} = \kappa_{\mathrm{D1}} \left(x_{\text{ORA}} \delta - x_{\text{DRA}} \right) + \kappa_{\mathrm{A2}} \left(\mu_{\mathrm{A}} x_{\text{DRO}} x_{\mathrm{A}} - x_{\text{DRA}} \right),  \end{array} $$

17g $$\begin{array}{*{20}l} {}&\frac{\mathrm{d}}{\mathrm{d} \tau}x_{\text{ARO}} = \kappa_{\mathrm{A1}} \left(x_{\text{ORO}} x_{\mathrm{A}} - x_{\text{ARO}} \right) + \kappa_{\mathrm{A2}} \left(x_{\text{ARA}} - \mu_{\mathrm{A}} x_{\text{ARO}} x_{\mathrm{A}} \right)  \\ {}& \hspace{16mm} + \kappa_{\mathrm{D2}}\left(x_{\text{ARD}} - \mu_{\mathrm{D}}x_{\text{ARO}} \delta \right), \end{array} $$

17h $$\begin{array}{*{20}l} {}& \frac{\mathrm{d}}{\mathrm{d} \tau}x_{\text{ORA}} = \kappa_{\mathrm{A2}} \left(\mu_{\mathrm{A}} x_{\text{ORO}} x_{\mathrm{A}} - x_{\text{ORA}} \right) + \kappa_{\mathrm{A1}} \left(x_{\text{ARA}} - x_{\text{ORA}} x_{\mathrm{A}} \right)  \\ {}& \hspace{16mm} + \kappa_{\mathrm{D1}} \left(x_{\text{DRA}} - x_{\text{ORA}} \delta \right), \end{array} $$

17i $$\begin{array}{*{20}l} {}& \frac{\mathrm{d}}{\mathrm{d} \tau}x_{\text{DRO}} = \kappa_{\mathrm{D1}} \left(x_{\text{ORO}} \delta - x_{\text{DRO}} \right) + \kappa_{\mathrm{A2}} \left(x_{\text{DRA}} - \mu_{\mathrm{A}} x_{\text{DRO}} x_{\mathrm{A}} \right)  \\ {}& \hspace{16mm} + \kappa_{\mathrm{D2}}\left(x_{\text{DRD}} - \mu_{\mathrm{D}} x_{\text{DRO}} \delta \right),  \end{array} $$

17j $$\begin{array}{*{20}l} {}& \frac{\mathrm{d}}{\mathrm{d} \tau}x_{\text{ORD}} = \kappa_{\mathrm{D2}} \left(\mu_{\mathrm{D}} x_{\text{ORO}} \delta - x_{\text{ORD}} \right) + \kappa_{\mathrm{A1}} \left(x_{\text{ARD}} - x_{\text{ORD}} x_{\mathrm{A}} \right)  \\ {}& \hspace{16mm} + \kappa_{\mathrm{D1}} \left(x_{\text{DRD}} - x_{\text{ORD}} \delta \right),  \end{array} $$

where *κ*_A1_,*κ*_A2_,*κ*_D1_ and *κ*_D2_ are the dimensionless parameters representing the rates of the both dissociation and association of the ligands given by

18 $$\begin{array}{*{20}l} \kappa_{\mathrm{A1}} := \frac{k_{\mathrm{dissA1}} }{ {k_{\text{decay}}} }, \quad \kappa_{\mathrm{A2}} := \frac{k_{\mathrm{dissA2}} }{ {k_{\text{decay}}} },  \\ \kappa_{\mathrm{D1}} := \frac{k_{\mathrm{dissD1}} }{ {k_{\text{decay}}} }, \quad \kappa_{\mathrm{D2}} := \frac{k_{\mathrm{dissD2}} }{ {k_{\text{decay}}} }, \end{array} $$

and *λ*_A1_,*λ*_A2_, and *μ*_A_are the dimensionless parameters representing affinities of ACh to the binding sites of the AChR given by

19 $$\begin{array}{*{20}l} \lambda_{\mathrm{A1}} := \frac{\mathrm{[\!R]}_{\text{total}}}{K_{\mathrm{A1}} }, \quad \lambda_{\mathrm{A2}} := \frac{\mathrm{[\!R]}_{\text{total}}}{K_{\mathrm{A2}} }, \quad \mu_{\mathrm{A}} := \frac{K_{\mathrm{A1}}}{K_{\mathrm{A2}}}, \quad \end{array} $$

with *K*_A2_:=*k*_dissA2_/*k*_assocA2_, and finally, *μ*_D_ and *δ* are the dimensionless parameters representing the site-selectivity and concentration of NDNB, respectively, given by

20 $$\begin{array}{*{20}l} \mu_{\mathrm{D}} := \frac{K_{\mathrm{D1}}}{K_{\mathrm{D2}}}, \quad \delta := \frac{{[\!\mathrm{D}]}}{ K_{\mathrm{D1}} }, \end{array} $$

with *K*_D1_:=*k*_dissD1_/*k*_assocD1_ and *K*_D2_:=*k*_dissD2_/*k*_assocD2_. Note that due to the scaling performed here, the number of parameters characterizing the properties of NDNB can be reduced from four to three: from (*k*_dissD1_,*k*_asoocD1_,*k*_dissD2_,*k*_assocD2_) to (*κ*_D1_,*κ*_D2_,*μ*_D_).

Furthermore, the order of the model can be reduced using the technique of singular perturbation [24, 25] based on an inherent multiple-timescale property of the model. Such a multi-scale property arises when the dissociation rate constants *k*_dissD1_ and *k*_dissD2_ of an NDNB are much smaller than other rate constants such as *k*_dissA1_,*k*_dissA2_ and *k*_decay_. By considering the limit *κ*_D1_, *κ*_D2_→0, the following equations hold from the Eq. 17:

21 $$\begin{array}{*{20}l}  & \frac{\mathrm{d}}{\mathrm{d} \tau}x_{\text{DRD}} = \frac{\mathrm{d}}{\mathrm{d} \tau}(x_{\text{ORD}}+x_{\text{ARD}}) = \frac{\mathrm{d}}{\mathrm{d} \tau}(x_{\text{DRO}}+ x_{\text{DRA}}) = 0. \end{array} $$

Thus, the values of *x*_DRD_,*x*_ORD_+*x*_ARD_ and *x*_DRO_+*x*_DRA_ do not change in the reduced-order model, or almost unchanged in the original model (3), from their initial values at *τ*=0. When the initial conditions are defined by the steady state concentrations under a given value of *δ*, the initial values of *x*_DRD_,*x*_ORD_,*x*_DRO_,*x*_ARD_ and *x*_DRA_ are given by

22a $$\begin{array}{*{20}l} x_{\text{DRD}}|_{\tau=0} &= O_{\text{DRD}}(\delta) := \frac{ \mu_{\mathrm{D}} \delta^{2} }{(1 + \delta)(1 + \mu_{\mathrm{D}} \delta) }, \end{array} $$

22b $$\begin{array}{*{20}l} x_{\text{ORD}}|_{\tau=0} &= O_{\text{ORD}}(\delta) := \frac{\mu_{\mathrm{D}} \delta }{ (1 + \delta)(1 + \mu_{\mathrm{D}} \delta)}, \end{array} $$

22c $$\begin{array}{*{20}l} x_{\text{DRO}}|_{\tau=0} &= O_{\text{DRO}}(\delta) := \frac{ \delta }{ (1 + \delta)(1 + \mu_{\mathrm{D}} \delta)}, \end{array} $$

22d $$\begin{array}{*{20}l} x_{\text{ARD}}|_{\tau=0} & = x_{\text{DRA}}|_{\tau=0} = 0 \end{array} $$

where *O*_DRD_,*O*_ORD_ and *O*_DRO_ stand for the fractions of the complex DRD, ORD, and DRO, respectively, at the steady state. Also, the total occupancy *O*_total_ of the AChRs by the NDNB is given by

23 $$\begin{array}{*{20}l}  O_{\text{total}}(\delta) := O_{\text{DRD}}(\delta) + O_{\text{ORD}}(\delta) + O_{\text{DRO}}(\delta). \end{array} $$

By using the equations from (21) to (23), the model (3) can be reduced to the following form:

24a $$\begin{array}{*{20}l} {}&\frac{\mathrm{d}}{\mathrm{d} \tau}x_{\mathrm{A}} = - x_{\mathrm{A}} +\kappa_{\mathrm{A1}}\lambda_{\mathrm{A1}} \{ x_{\text{ARA}} - x_{\mathrm{A}} (O_{\text{ORD}}(\delta) - x_{\text{ARD}}) \}  \\ {}& \hspace{16mm} +\kappa_{\mathrm{A2}}\lambda_{\mathrm{A2}} \{ x_{\text{ARA}} - x_{\mathrm{A}} (O_{\text{DRO}}(\delta) - x_{\text{DRA}}) \}  \\ {}& \hspace{16mm} +\kappa_{\mathrm{A1}}\lambda_{\mathrm{A1}} \{ x_{\text{ARD}} + x_{\text{ARO}} - x_{\mathrm{A}} (1-x_{\text{ARA}}-x_{\text{ARO}} - O_{\text{total}}(\delta)) \}  \\ {}& \hspace{16mm} +\kappa_{\mathrm{A2}}\lambda_{\mathrm{A2}} \{ x_{\text{DRA}}+ x_{\text{ORA}} - x_{\mathrm{A}} (1-x_{\text{ARA}} -x_{\text{ORA}}- O_{\text{total}}(\delta)) \}, \end{array} $$

24b $$\begin{array}{*{20}l} {}& {\frac{\mathrm{d}}{\mathrm{d} \tau}x^{\ast}_{\text{ARA}} = - \kappa_{\text{close}} x^{\ast}_{\text{ARA}} + \kappa_{\text{open}}x_{\text{ARA}},} \end{array} $$

24c $$\begin{array}{*{20}l} {}& \frac{\mathrm{d}}{\mathrm{d} \tau}x_{\text{ARA}} = \kappa_{\mathrm{A1}} \left(x_{\text{ORA}} x_{\mathrm{A}} - x_{\text{ARA}} \right) + \kappa_{\mathrm{A2}} \left(\mu_{\mathrm{A}} x_{\text{ARO}} x_{\mathrm{A}} - x_{\text{ARA}} \right)  \\ {}& \hspace{16mm} {+ \kappa_{\text{close}} x^{\ast}_{\text{ARA}} - \kappa_{\text{open}}x_{\text{ARA}}}, \end{array} $$

24d $$\begin{array}{*{20}l} {}& \frac{\mathrm{d}}{\mathrm{d} \tau}x_{\text{ARO}} = \kappa_{\mathrm{A1}} \left\{ x_{\mathrm{A}} (1-x_{\text{ARA}}-x_{\text{ARO}}-x_{\text{ORA}}- O_{\text{total}}(\delta))- x_{\text{ARO}} \right\}  \\ {}& \hspace{16mm} +\kappa_{\mathrm{A2}} \left(x_{\text{ARA}} - \mu_{\mathrm{A}} x_{\text{ARO}} x_{\mathrm{A}} \right), \end{array} $$

24e $$\begin{array}{*{20}l} {}& \frac{\mathrm{d}}{\mathrm{d} \tau}x_{\text{ORA}} = \kappa_{\mathrm{A2}} \left\{ \mu_{\mathrm{A}} x_{\mathrm{A}} (1-x_{\text{ARA}}-x_{\text{ARO}}-x_{\text{ORA}}- O_{\text{total}}(\delta)) - x_{\text{ORA}} \right\}  \\ {}&\hspace{16mm} + \kappa_{\mathrm{A1}} \left(x_{\text{ARA}} - x_{\text{ORA}} x_{\mathrm{A}} \right), \end{array} $$

24f $$\begin{array}{*{20}l} {}& \frac{\mathrm{d}}{\mathrm{d} \tau}x_{\text{ARD}} = \kappa_{\mathrm{A1}} \left\{ x_{\mathrm{A}} (O_{\text{ORD}}(\delta) - x_{\text{ARD}}) - x_{\text{ARD}} \right\}, \end{array} $$

24g $$\begin{array}{*{20}l} {}& \frac{\mathrm{d}}{\mathrm{d} \tau}x_{\text{DRA}}= \kappa_{\mathrm{A2}} \left\{ \mu_{\mathrm{A}} x_{\mathrm{A}} (O_{\text{DRO}}(\delta) - x_{\text{DRA}}) - x_{\text{DRA}} \right\}. \end{array} $$

## Data Availability

The data generated and/or analyzed in this research can be reproduced using numerical simulations explained in Methods section.

## Abbreviations

NDNB: Nondepolarizing neuromuscular blocking drugs
ACh: Acetylcholine
AChR: Acetylcholine receptor
